# Leadership in Orchestra Emerges from the Causal Relationships of Movement Kinematics

**DOI:** 10.1371/journal.pone.0035757

**Published:** 2012-05-09

**Authors:** Alessandro D'Ausilio, Leonardo Badino, Yi Li, Sera Tokay, Laila Craighero, Rosario Canto, Yiannis Aloimonos, Luciano Fadiga

**Affiliations:** 1 Robotics, Brain and Cognitive Sciences, Istituto Italiano di Tecnologia, Genova, Italy; 2 Department of Computer Science, University of Maryland, College Park, Maryland, United States of America; 3 Şişli Symphony Orchestra, Istanbul, Turkey; 4 Philarmonie de Chambre Tokay, Paris, France; 5 DSBTA, Università di Ferrara, Ferrara, Italy; French National Centre for Scientific Research, France

## Abstract

Non-verbal communication enables efficient transfer of information among people. In this context, classic orchestras are a remarkable instance of interaction and communication aimed at a common aesthetic goal: musicians train for years in order to acquire and share a non-linguistic framework for sensorimotor communication. To this end, we recorded violinists' and conductors' movement kinematics during execution of Mozart pieces, searching for causal relationships among musicians by using the Granger Causality method (GC). We show that the increase of conductor-to-musicians influence, together with the reduction of musician-to-musician coordination (an index of successful leadership) goes in parallel with quality of execution, as assessed by musical experts' judgments. Rigorous quantification of sensorimotor communication efficacy has always been complicated and affected by rather vague qualitative methodologies. Here we propose that the analysis of motor behavior provides a potentially interesting tool to approach the rather intangible concept of aesthetic quality of music and visual communication efficacy.

## Introduction

Coordinated action is one of the basic abilities for social interaction. This skill is at the basis of evolutionarily relevant collective behaviors such as defense, reproduction, or hunting [1]–[4]. Coordinated action, in humans, has been formalized in many ways and constitutes one of the frontiers of cognitive neuroscience [5], [6]. Generally speaking, coordinated action might be conceived as a successful degree of synchrony/complementarity between actions performed by at least two individuals [7]. Whereas, joint action require the sharing of the same goal and does not necessarily requires a specific motor coordination among all the agents. Both action coordination and joint action requires the continuous exchange of information to allow understanding and prediction of other's motor intentions. Research indicates that in both monkeys [8] and humans [9], [10] the motor system is recruited during this information exchange, which can be considered a sort of sensorimotor communication [11]. Therefore, sensorimotor communication is the accurate negotiation of our own motor output according to sensorimotor messages sent by other participants in the interaction.

In this context, music orchestras are a particularly interesting instance of sensorimotor communication between several players and a conductor. As a matter of fact, ensemble music performance is also a remarkable instance of social interaction in which the conductor uses her/his motor behavior to drive the players toward a common aesthetic goal (joint action). Thus, such a scenario is naturally suited for the study of non-verbal communication flows, since movement coordination is a skill musicians train for years. More specifically such coordination, at the individual level, can be modeled as a computation transforming salient sensory information (sensory representation of others' action kinematics) into motor control parameters [12], [13]. However, a rigorous testing of inter-individual coordination in such an ecological scenario poses a series of technical challenges, mainly related to data acquisition and analyses.

**Figure 1:**
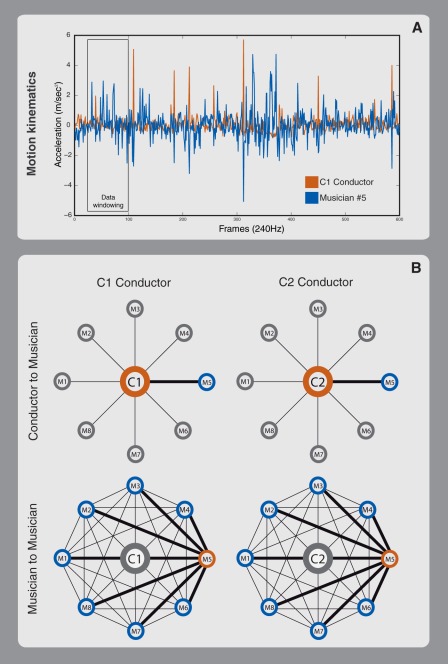
Experimental design. For each piece of music, the time is divided into overlapping windows (panel A show acceleration profiles of C1 conductor in orange and Violin number 5 in blue). The pairwise driving forces between the conductor (orange circle) and each player (blue circle) are computed. The performances are evaluated using conductor-player pairs. Subsequently, compute the pairwise driving forces between players. The summed value of forces between each player (in orange) towards all other musicians (blue circle), excluding the contribution of the conductor (here in grey), forms the interaction strength for that musician (Panel B).

In our experiments we applied GC method to musicians' and conductors' kinematic data. Granger causality is a statistical concept of causality that is based on prediction. According to Granger causality, if a signal X1 "Granger-causes" a signal X2, then past values of X1 should contain information that helps predict X2 above and beyond the information contained in past values of X2 alone. Its mathematical formulation is based on linear regression modeling of stochastic processes [14], [15]. In the present study, we explored whether conductors' kinematics were associated to a differential influence on musician's performance (driving force) and if this was able to affect inter-musician interaction (interaction strength; see Figure 1).

Eight violin players played five well-known pieces of music with two orchestra conductors (C1, C2). Pieces were selected because they were especially suitable to differentiate the talents and capacities of conductors. Musicians' and conductors' kinematic data acquisition was carried out with an infrared optical system with passive markers placed on the upper end of players' bows (one marker per bow) and conductors' final tip of the baton (one per baton). Furthermore, we had expert musicians rate (offline and blind to the scope of the experiment) audio recordings on several subjective scales, such as their ability to follow the piece (separately for melody and rhythm), the degree of musical entrainment and that of emotional involvement. Our aim was to investigate whether we could derive: 1) the amount of driving influence exerted by the conductor on the players; 2) the degree of sensorimotor communication among musicians. Furthermore, these parameters were associated to expert judgments of musical performance to assess a possible qualitative relation between sensorimotor communication and the overall perceived quality of musical execution.

## Results

**Figure 2:**
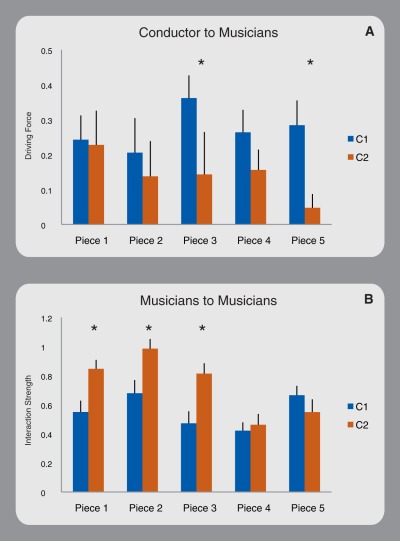
Conductor-to-musician, musician-to-musician communication. Conductor to musicians driving force (A), musician-to-musician interaction strength (B) are evaluated across musical pieces and shown in the upper two histograms. Bars show the standard error of the mean and asterisks denote significant differences.

Conductors' average driving force towards musicians was significantly different in two of the pieces (3, and 5; factor Conductor: F(1,7) = 48.78-p<0.0005; factor Piece: F(4,28) = 1.89-p>0.05; Interaction: F(4,28) = 4.63-p<0.01; Figure 2a), whereas the conductors modulated inter-musician average interaction strength in three pieces (1, 2 and 3; factor Conductor: F(1,7) = 58.75-p<0.0005; factor Piece: F(4,28) = 92.85-p<0.0001; Interaction: F(4,28) = 19.37-p<0.0001). Here, we quantitatively show the Granger causality pattern among conductors and musicians as a sensorimotor conversation between several individuals: musicians accommodate their performance according to non-linguistic motor messages received from other musicians and from the conductors.

**Figure 3:**
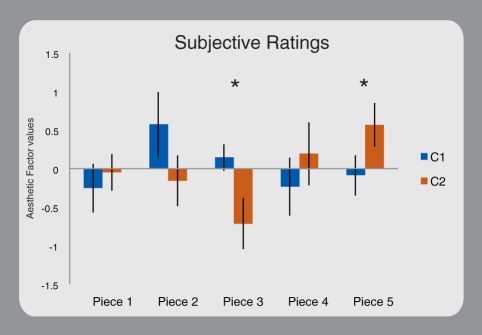
Subjective ratings. The histogram represents the Aesthetic factor (F1) modulation across pieces and conductors. Evaluations were performed by other musicians on the musical audio recordings. Bars show the standard error of the mean and asterisks denote significant differences.

Expert judgments were modulated by piece and conductor (Figure 3). Specifically, two pieces (3 and 5; factor Conductor: F(1,9) = 0.14; p = 0.71; p_adj_ = 0.71; factor Piece: F(4,36) = 1.79; p = 0.15; p_adj_ = 0.17; Interaction: F(4,36) = 4.17; p = 0.007; p_adj_ = 0.028) were considered significantly different between the two conductors. Piece number 3 was the only one where the increased influence of the C1 conductor was paralleled by a significant reduction in inter-musician influences. Subjective ratings showed larger perceived quality with C1. In piece number 5, C1 conductor exerted an increased drive that was not paralleled by a reduction in inter-musician influences. Ratings favored the C2 conductor.

## Discussion

Aesthetic appreciation is an intriguing human capacity and yet one of the most intangible aspects of higher cognition. However, exploring the rules governing such experience has potentially a great relevance for neuroscience [16]. In fact, the arts may be fruitfully exploited to study brain mechanisms since according to Zeki and Lamb [17] "[visual] artists are unknowingly exploring the organization of the visual brain though with techniques unique to them". Music, in this framework, might be used as a window into other complex integrative brain processes. On one hand a composer might be probing complex visuo-spatial processes, as for example in Bach's Canons of the Musical Offering. On the other hand, music live performance might impinge on the listeners' sensorimotor integration capabilities and inter-musician interaction processes. Indeed, bodily movements together with auditory information are integrated, in the listeners, to produce the rewarding experience of a musical appreciation. Orchestras may thus form the perfect model of complex non-verbal social interaction and social aesthetics.

In such context, here we described the complex pattern of sensorimotor communication in the orchestra scenario as well as the parallel effect of such interaction on the perceived quality of the musical output. We showed that the two conductors exhibited different driving-force strengths towards musicians in some pieces (3 and 5), whereas communication strength among players was also modulated by the characteristics of the two conductors in others (1, 2 and 3). Even if the differences between conductors may be attributed to conducting style or expertise we discovered a significant modulation of the whole network of interactions across pieces. On the other hand, Granger's method may be misled by latent un-known variables. In the orchestra example we know we have a latent variable that is the score. The score is the ultimate origin of both conductor and musicians behaviors. However, the score is kept constant across conductors thus cancelling its effects.

The dynamical network of interactions is naturally aimed at producing a pleasant effect in the listeners. In this respect, music is a complex and formalized sensorimotor task whose goal is to induce states (in the listeners) that go beyond any straightforward quantification, as is the case of aesthetic appreciation. We might say that the only measure of sensorimotor communication efficacy is the aesthetic quality of music. Interestingly enough, aesthetic appreciation of music orchestras' performance was associated to the concurrent increase of conductor-to-musician influence and a reduction of musician-to-musician information flow. Instead the simple increase in conduction drive might be detrimental to perceived quality if this is not followed by a reduced inter-musician interaction.

The mechanism that characterizes a successful sensorimotor conversation requires that all participants are able to send and receive subtle messages in the form of visual motor gestures and auditory events. This encoding/decoding process might be conceived of as a complex and hierarchical input-output mapping ranging from rote sensorimotor mapping to the highest level of human action organization [13], [18]. In this context, a mirror-like fronto-parietal circuit, due to its properties, might be particularly important in non-verbal communication between individuals [19], [20]. Action mirroring, however, does not facilitate coordinated action *per se*, in fact, coordination may often require the execution of different/complementary actions between participants [5]. Recently however, it has been demonstrated that human mirror neuron mechanisms might be tuned for action coordination rather than simple action mirroring [21]. These results are in line with an interactive account of mirror-like activities forming the basis sensorimotor communication.

However, sensorimotor communication cannot be the result of purely reactive mechanisms. A reactive mechanism uses sensory data to plan and execute the appropriate motor reaction. This is not feasible in motor control since delays in feedback are too long for an efficient and smooth motor execution [22], [23]. Motor control theory introduced the idea of (internal) models that associate a given command to an expected (surrogate) sensory feedback [12]. In our view, sensorimotor communication relies on a model of the information sent by the other participant rather than on actual sensory data. Real data can only later be integrated for model correction/learning. In this context, a mirror-like mechanism is probably the best candidate to perform such modeling [24], [25]. Each musician may build a model of the conductor (and musicians) behavior to anticipate/simulate the conductor's (or musicians') movements. This modeling ability might indeed be at the basis of musical expertise. In fact, beside technical skills, a successful orchestra might be the results of players and conductors that have built efficient and reliable models of others' action.

Other's action modeling must contain all relevant information sent by the participants. The conductor is certainly communicating low level features such as attacks timing as well as higher-level interpretational aspects such as supra-segmental information. Specifically, we believe that in orchestras there is both sensorimotor coordination and joint action. In some parts of a piece, the violins may have to play unison thus requiring accurate low-level sensorimotor coordination. However, probably on longer temporal scales, the shared goal of the orchestra goes beyond the perfect execution of a technical passage but rather it is concerned with the specific interpretation of a musical piece. Furthermore, communication is not unidirectional and the conductors receive continuous feedback from the musicians just like musicians are heavily influenced by other musicians in their section. In fact, the orchestra scenario is a particularly interesting case since it is characterized by two qualitatively different kinds of communication. Conductor to musicians and musician to musician interactions are indeed radically different because of the role played by the participants (leader Vs follower) and by the different kind of movements that are executed. Differences may also be related to the saliency of kinematic feature and thus the granularity of sensori-motor mirroring (subtle movement features as opposed to larger scale interpretational cues). Also, these processes may heavily interact with the specificity of the musical passage that is being performed. The weighting of these processes may ultimately affect perceived quality. Although the present data-set doesn't enable any quantitative evaluation of this hypothesis, we could show that the orchestra scenario offers a prototypical situation to study these extremely interesting processes.

In conclusion, the present study adds significant data to the growing body of research that considers musicians as a model to study sensorimotor brain plasticity and organization [26]. Here, we used musicians as a model of how effective sensorimotor communication might be, based on efficient gesture coordination. In fact, each musician has a score, is well trained on the pieces s/he is playing, and can listen and see what other musicians do. However, the violinist has to concurrently follow the conductor that provides critical information on how to interpret a given phrase. Therefore, musicians have to build efficient expectations regarding several sources of information and mix them up in order to reach the required performance. Among all sources, the conductor might impose a sort of supra-segmental layer affecting the emotional entrainment and, ultimately, the aesthetic quality for the listeners. Following these lines, we propose that the conductor will significantly change the perceived quality of a piece when s/he both increases his/her influence on musicians and, at the same time, expresses a personality able to overshadow the inter-musician communication. In simpler terms, this might be the essence of leadership. In conclusion, here we could quantify the non-verbal communication patterns among musicians and conductor that may affects the rather intangible concept of aesthetic quality of music.

## Methods

### Orchestra data acquisition

Eight violin players of the “Città di Ferrara” Orchestra participated in the study. Two orchestra conductors conducted the violinists (C1, C2). Written informed consent was obtained from all participants. The Ethical Committee of University of Ferrara approved all the procedures.

Musicians played five pieces they knew and rehearsed several times. Each piece was repeated three times. Therefore, we recorded data for thirty musical exerpts, 15 for each conductor. These pieces were selected because, despite their apparent simplicity, they contain several changes in tempo, and because orchestras are usually over-trained on them. Mozart symphony No. 40 is a work especially suitable to differentiate the talents and capacities of conductors. Indeed, it's a 2-stroke tempo, which can still be beaten at a time, allows acceleration in tempo ad libitum if beats are made with sufficient advance. While as usual, in concert, these tempo changes meet the conductor's expressive intentions, in the context of our experience they were simply intended to put to the test the respective ability to anticipate of both conductors. In addition, the passage chosen in this work of Mozart includes such significant ends of phrases and beginnings of phrases as are needed to reveal the skill, or lack of it, of the conductor in the imposition of a style (baroque, romantic, etc.) and of a musical character (forte, piano, staccato, agitato, etc.) as well as its authority over the musicians in sync bowing. Audio recordings were performed using a professional ambient microphone (AKG, C1000s), sampled at 44.1 kHz and digitally recorded on a computer for further analysis.

Kinematic data acquisition was carried out with a Qualysis system (Qualisys Medical AB, Gothenburg, Sweden) with three cameras recording the 3D (absolute) position of passive markers placed on the upper end of players' bows (one marker per bow) and conductors' final tip of the baton (one per baton). Data was acquired at a sampling rate of 240 Hz and stored for offline analysis.

### Data preprocessing and analyses

We first used the spline method [27] to handle the missing data in the 3D trajectories. The spline method interpolates the data with continuous third order derivatives. The missing data is due to the fact that a sensor might not be visible to some cameras when it is out of range. We then computed the magnitude of the acceleration from each 3D trajectory. There is a two-fold motivation behind this choice. First, Granger-causality requires the time-series to be covariance-stationary. In our study the trajectories were non-stationary so we differentiated the signal (as it is common practice) to obtain a stationary signal. Both first and second order derivatives (acceleration) turned out to be stationary (within the observation windows, see below). Second, we believe that, in terms of transfer of information (concerning musical expressiveness) between conductor and violinists and among violinists, accelerations of bows and baton are more informative than their trajectories and velocities (also suggested by author S.T.).

Finally the (magnitude of) acceleration time-series were demeaned, detrended, normalized (to z-scores) and windowed into overlapping windows (2/3 of a window overlapped with the adjacent windows). Granger causality inference was carried out at each window. In order to assess whether the window length affected the causal relations inference, three different window sizes were used: 1, 5 and 10 seconds. No low-pass filtering was applied to the signal, as it could have introduced artifacts in the inference of Granger causality relations [28].

It is worth mentioning that GC is not a measure of true causality, just like any statistical method that tries to infer causality from observed data. Indeed statistical associations on observed data do not logically imply causation [29]. True causality can only be inferred when variables can be directly controlled (i.e. when a perturbation to the system is applied or variables are directly manipulated rather than observed).

### Experiment 1

The aim of this experiment was to compare the “driving forces” of the two conductors on each violinist. At each observation window we tested whether the Granger causality values in each conductor-violinist pair were statistically significant. Note that according to our definition of driving force (see Methods S1) the conductor's driving force mainly depends on the number of times the conductor significantly exerts his influence on the violinist rather than on the magnitude of the conductor's influence. Finally, in this experiment and in experiment 2 (see below) the Auto-Regressive (AR) model order was set to 10 (see Methods S1 for details on the method used to select the model order).

The proportion of times that the conductor significantly influenced any given musician was averaged across the three repetitions of each piece. Preprocessed data, after a check for the necessary statistical assumptions, was tested using a two-way repeated measures ANOVA (RM-ANOVA) including factors Piece (1, 2, 3, 4, 5) and Conductor (C1, C2). Tukey HSD post-hoc tests were used to test for significant pair-wise comparisons.

### Experiment 2

The aim of this experiment was to investigate whether the “interaction strength” among violinists was dependent on the conductor. Note that in this case the use of the Conditional Granger causality is mandatory. Using a non-conditional Granger causality would mean ignoring the influence of the conductor and misinterpret it as influence from one musician to the other. So, e.g., simple delays between two violinists would be erroneously interpreted as causal relations. First we summed the proportions of times that one musician is significantly causing all other musicians, to measure the total driving force exerted towards the other participants. Then this value was averaged across the three repetitions of each piece. Preprocessed data, after a check for the necessary statistical assumptions, was tested using a two-way RM-ANOVA including factors Piece (1, 2, 3, 4, 5) and Conductor (C1, C2). Tukey HSD post-hoc tests were used to test for significant pair-wise comparisons.

### Aesthetic evaluation experiment

In a second session, ten musicians (mean age: 33±9.4 STD; years of formal training: 7.9±3.6 STD; years played: 14.9±9.1 STD; start age: 8.5±2.4 STD; hours of practice per week: 7.8±4.8 STD) who did not participate in the first study were asked to rate the audio tracks recorded previously. They had to fill a web-based questionnaire, including an initial part investigating their level of musical expertise and a second one for the evaluations of audio tracks. They listened to the thirty musical exerpts (length ranging from 50 seconds to 2 minutes) and after each piece they had to answer to a series of questions regarding it. The questionnaire included 8 questions regarding different domains and specifically: i) how well they could concentrate on the pieces, ii) on the melody, iii) on the tempo, iv) on the rhythm, v) how much they felt transported by the piece, vi) how much the felt they were simulating playing, vii) how emotional was the piece, viii) and how well the piece was performed (See Methods S1 and Figure S1). For each question they had to move a visual continuous slider ranging from "Low" to "High". Slider position could be a value ranging from 0 to 100. Stimuli presentation was random and both presentation and response collection was done via the same web-based interface. The whole experiment lasted 45–50 minutes. Since the questionnaire was investigating highly correlated psychological dimensions, we ran a factor analysis on the eight items to extract the main components of variance. Further inferential statistics were run for the extracted factors' score matrices. However, data sphericity was not met and we could not proceed with standard RM-ANOVA. Instead we both used a multivariate approach (Wilks' Lambda) as well as a Greenhouse-Geisser corrected RM-ANOVA. In both cases we used factors Piece (1, 2, 3, 4, 5) and Conductor (C1, C2). Significant factors and interactions were further explored via paired t-tests (Bonferroni correction).

## Supporting Information

Figure S1 **Factor loadings for the subjective questionnaire items.** X and Y axes show loading on the two factors extracted with factor analysis. Q1 to Q8 represent loadings on the two factors for each of the eight questions.
(EPS)

Methods S1 **Additional details on the application of Granger causality method as well as more details on the analyses and results.**
(DOCX)
