# Supplementary material for: Leadership in Orchestra Emerges from the Causal Relationships of Movement Kinematics
Source: PLoS One. 2012 May 9;7(5):e35757. doi: 10.1371/journal.pone.0035757 (PMC3348913; doi:10.1371/journal.pone.0035757)
Supplement: Methods S1 — Additional details on the application of Granger causality method as well as more details on the analyses and results. (DOCX) [file pone.0035757.s002.docx]

**Leadership in orchestra emerges from the causal relationships of movement kinematics**

**Methods**

*Modeling human motion using linear predictors*

Granger causality, in its standard and linear formulation, is based on (linear) Autoregressive Models (AR). AR models belong to the family of the Linear Dynamical Systems, which has been extensively used in modeling human motion [30-33]. An AR(k) model of a time series $x$ is defined as:

$$\left( 1 \right) x\left( t \right)= \sum_{j=1}^{l} a_{j}x\left( t-j \right) + \varepsilon_{R}\left( t \right)$$

where $x(t)$ is the value of the time series $x$ at time $t$, $l$ is the order of the model (i.e., the length of the history observed in the model), $a_{j}$ ($j=1, \ldots, l$) are the weights for the history (the model parameters), and $\varepsilon_{R}(t)$ is the residual (prediction error).

There are two widely used criteria for selecting the optimal order of a linear predictor (i.e., the order that guarantees the best goodness of fit of the model): the Akaike’s Information Criterion (AIC) [34] and the Schwarz’s Bayesian Information Criterion (BIC) [35]. The parameters $a_{j}$ can be computed by using Ordinary Least Squares.

Since Granger causality is based on AR models the validity of the inferred causal relations depends on the validity of the AR models (more specifically of the unrestricted AR models, see next section). To assess the validity of an AR model different tests can be carried out, ranging from tests of the non-correlation of the residuals to tests of the goodness-of-fit of the model (for example, the goodness-of-fit can be measured as the sum of squares of the residuals).

*Granger causality*

A time series X is said to “Granger cause” a time series Y, if the past values of X provide statistically significant information to predict the next value of Y [14]. The prediction is computed using AR models. Two AR models are required: an unrestricted AR model where the history of all time series is assumed to contribute to the prediction of the current value of a time series; and a restricted AR model where the time series whose causality value (on the other time series) is computed is excluded from the history. Given two time series X and Y, the unrestricted model is defined as:

$x\left( t \right)= \sum_{j=1}^{l} a_{U,j}x\left( t-j \right)+ \sum_{j=1}^{l} b_{U,j}y\left( t-j \right)+ \varepsilon_{U}\left( t \right)$

(2)

$y\left( t \right)= \sum_{j=1}^{l} c_{U,j}x\left( t-j \right)+ \sum_{j=1}^{l} d_{U,j}y\left( t-j \right)+ \eta_{U}(t)$

While the restricted model is defined as:

$x\left( t \right)= \sum_{j=1}^{l} a_{R,j}x\left( t-j \right) + \varepsilon_{R}\left( t \right)$

(3)

$y\left( t \right)= \sum_{j=1}^{l} d_{R,j}y\left( t-j \right) + \eta_{R}(t)$

Then the magnitude of the causality from X to Y and from Y to X can be measured respectively as:

$(4) \mathcal{F}_{x\to y}= \ln\frac{H_{R}}{H_{U}}$, $\mathcal{F}_{y\to x}= \ln\frac{E_{R}}{E_{U}}$

where E and H are the model error variances:

${\left( 5 \right) E}_{R}=var\left( \varepsilon_{R}\left( t \right) \right)$, $E_{U}=var\left( \varepsilon_{U}\left( t \right) \right)$,

$H_{R}=var\left( \eta_{R}\left( t \right) \right), H_{U}=var\left( \eta_{U}\left( t \right) \right)$

Once the Granger causality values have been computed, we need to test their statistical significance, i.e., we need to infer the significant causal relations. A significance test can be done by carrying out an F-test of the null hypothesis that the model parameters referring to the time series of which we compute the “causal strength” (on the other time series) are all zero (e.g., parameters $b_{U,j}$ in model (2) to test the significance of $\mathcal{F}_{y\to x}$). When more than two time series are analyzed some corrections (e.g., the Bonferroni correction) are applied to the F-test.

When the interaction of more than two time series is addressed, repeated pair-wise Granger causality computations can lead to misleading results. To avoid that, a simple extension of Granger causality, sometimes referred to as Conditional Granger causality, has been proposed by Ding et al., 2006 [36]. Suppose we have three time series X, Y and Z, then the Conditional Granger causality from Y to X given Z is defined as the log ratio of the error variance of the restricted model where only Y is excluded from the history (when modeling X) and the variance of the unrestricted model, where the history of all time series X, Y and Z is included.

The Granger causality analysis, including AR model validation and statistical tests of causal interactions, were carried out by using the “Granger Causality Connectivity Analysis” MatLab toolbox [28].

*Non-linear Granger causality*

Granger causal relations might be erroneously inferred (or ignored) when the linearity assumptions of the linear AR models are wrong, i.e., when there are significant non-linear interdependencies between the observed times series. Several solutions to extend Granger causality to the non-linear case have been proposed [37, 38]. We implemented the non-linear Granger causality method proposed by Ancona et al., 2004 [39] and based on kernel-based AR models where kernels are radial basis functions. Unexpectedly, on our dataset the non-linear AR models turned out to produce a much poorer goodness-of-fit (measured as sum of squares of the residuals) than the linear AR models for all the configurations of free parameters (i.e., number of radial basis functions and their standard deviation) we tried. As a consequence all results reported in the paper only refer to the linear Granger causality analysis.

A possible cause of poor goodness-of-fit of the non-linear model could be the fact that the subset of configurations of the free parameters where we searched for “optimal” configurations did not contain any good-enough configurations. For example the number of radial basis functions we tried (up to 100) may have been too small for the time series we analyzed.

*Experiment 1*

The aim of this experiment was to compare the “driving forces” of the two conductors on each violinist. At each observation window we tested whether the Granger causality values in each conductor-violinist pair were statistically significant. Then we computed the driving force, in a given piece of music, of the conductor (C) on violinist $V_{i}$ as:

$(6) \frac{\sum_{j}^{N_{w}} {S\mathcal{(F}}_{C_{j}\to V_{i,j}}{)- S\mathcal{(F}}_{V_{i,j}\to C_{j}})}{N_{w}}$

where the $C_{j}$ and $V_{i,j}$ are respectively the slices of the conductor and of the $V_{i}$ violinist time series at window j, $N_{w}$ is the overall number of observation windows and

$\left( 7 \right) {S\mathcal{(F}}_{x\to y})= \left\{ \begin{aligned} 1 if \mathcal{F}_{x\to y}is significant \\ 0 otherwise \end{aligned} \right.$

As mentioned above, a free parameter of the analysis we carried out was the length of the observation. Different window sizes result in different $N_{w}$ values and different driving force values. The results reported are obtained with a 5-second window, as this window size was the size that produced the best goodness of fit (in terms of average squared residuals) of the (unrestricted) AR model.

Note that according to our definition the conductor’s driving force mainly depends on the number of times the conductor significantly exerts his influence on the violinist rather than on the magnitude of the conductor’s influence. Finally, in this experiment and in experiment 2 (see below) the AR model order was fixed and set to 10. A constant model order was used to guarantee that the autoregressive models relied on the same ‘history’ independently of the conductor so that no bias due to different histories could affect the comparison between the two conductors. The fixed model order was selected using the following method. We first used AIC and BIC to compute model orders for each observation window (within each execution). At each observation window the two model orders selected by the AIC and the BIC criterions are the orders that minimize AIC and BIC respectively. The average model order (averaged over each piece of music) ranged between 2 and 10. The highest value (10) was then chosen as the fixed model order used for all the autoregressive models. Within the 2-10 range, the maximum value 10 is the one by which the complexity of the model is largest (and so, in theory, its goodness of fit on the training data) and it is motivated by the fact that by using that value we never miss any relevant history at any observation window (assuming that AIC and BIC capture all and only the relevant history). When comparing the different model orders in the 2-10 range the model order 10 produced the best goodness of fit, as expected.

The proportion of times that the conductor significantly influenced any given musician was averaged across the three repetitions of each piece. Preprocessed data, after a check for the necessary statistical assumptions, was tested using a two-way repeated measures ANOVA (RM-ANOVA) including factors Piece (1, 2, 3, 4, 5) and Conductor (C1, C2). Tukey HSD post-hoc tests were used to test for significant pair-wise comparisons.

*Experiment 2*

The aim of this experiment was to investigate whether the “interaction strength” among violinists was dependent on the conductor. The interaction strength was defined as:

$\left( 8 \right)$ $\frac{\sum_{k}^{N_{w}} \sum_{i} \sum_{i\neq j} {S\mathcal{(F}}_{V_{i,k}\to V_{j,k}| C})}{{(N}_{w}/(N_{v}\left( N_{v}- 1 \right))}$

where $N_{v}$ is the number of violinists and $N_{v}(N_{v}- 1)$ is a normalization term. Note that in this case the use of the Conditional Granger causality is mandatory. Using a non-conditional Granger causality would mean ignoring the influence of the conductor and misinterpret it as influence from one musician to the other, so, e.g., simple delays between two violinists would be erroneously interpreted as causal relations.

After computing the interaction strength at each piece (using equation (8)) we averaged it across the three repetitions of each piece. Then this value was averaged across the three repetitions of each piece. Preprocessed data, after a check for the necessary statistical assumptions, was tested using a two-way RM-ANOVA including factors Piece (1, 2, 3, 4, 5) and Conductor (C1, C2). Tukey HSD post-hoc tests were used to test for significant pair-wise comparisons.

*Aesthetic evaluation experiment*

In a second session, ten musicians (mean age: 33 ± 9.4 STD; years of formal training: 7.9 ± 3.6 STD; years played: 14.9 ± 9.1 STD; start age: 8.5 ± 2.4 STD; hours of practice per week: 7.8 ± 4.8 STD) who did not participate in the first study were asked to rate the audio tracks recorded previously. They had to fill a web-based questionnaire, including an initial part investigating their level of musical expertise and a second one for the evaluations of audio tracks. They listened to the thirty musical excerpts (length ranging from 50 seconds to 2 minutes) and after each piece they had to answer to a series of questions regarding it. The questionnaire included 8 questions regarding different domains and specifically: i) how well they could concentrate on the pieces, ii) on the melody, iii) on the tempo, iv) on the rhythm, v) how much they felt transported by the piece, vi) how much the felt they were simulating playing, vii) how emotional was the piece, viii) and how well the piece was performed. For each question they had to move a visual continuous slider ranging from "Low" to "High". Slider position could be a value ranging from 0 to 100. Stimuli presentation was random and both presentation and response collection was done via the same web-based interface. The whole experiment lasted 45-50 minutes. Since the questionnaire was investigating highly correlated psychological dimensions, we ran a factor analysis on the eight items to extract the main components of variance. Further inferential statistics were run for the extracted factors’ score matrices. However, data sphericity was not met and we could not proceed with standard RM-ANOVA. Instead we both used a multivariate approach (Wilks’ Lambda) as well as a Greenhouse-Geisser corrected RM-ANOVA. In both cases we used factors Piece (1, 2, 3, 4, 5) and Conductor (C1, C2). Significant factors and interactions were further explored via paired t-tests (Bonferroni correction).

**Additional Results**

Results of GC analyses showed that the causal influence between conductors and each player was different across conductors. The C1 conductor drives the orchestra in two pieces out of five - namely piece 3 (p=0.001) and 5 (p=0.0006) (factor Conductor: F(1,7) = 48.78 - p<0.0005; factor Piece: F(4,28) = 1.89 - p>0.05; Interaction: F(4,28) = 4.63 - p<0.01). Moreover, we also found that players show a different pattern of driving forces between themselves when they play under the direction of the two conductors. Under the direction of the C1 conductor, each player has less inﬂuence on the other players in three pieces out of five - namely piece 1 (p=0.0002), 2 (p=0.0002) and 3 (p=0.0002) (factor Conductor: F(1,7) = 58.75 - p<0.0005; factor Piece: F(4,28) = 92.85 - p<0.0001; Interaction: F(4,28) = 19.37 - p<0.0001).

The factor analysis (Varimax rotation) on the questionnaire items revealed 2 orthogonal factors (F1 and F2) significantly fitting the data (Maximized log-likelihood value= -0.3172; chi-square [dfe=13] = 29.87; p = 0.0049). The estimated loading of each factor by each questionnaire item showed a clear pattern (Question I: F1=0.2155, F2=0.5556; II: F1=0.1940, F2=0.1147; III: F1=0.0598, F2=0.3570; IV: F1=-0.1500, F2=0.8154; V: F1=0.8853, F2=0.4389; VI: F1=0.5215, F2=-0.1286; VII: F1=0.7910, F2=0.4366; VIII: F1=0.6625, F2=-0.0684; Figure S1). The factor loading-matrix gives us a description of the underlying meaning of each factor. Factor 1 is mainly loaded by question V, VII, VIII and VI, whereas Factor 2 is mainly loaded by question IV, I and III. Therefore, Factor 1 seems to reflect more aesthetic judgments devoid of any technical evaluation, whereas Factor 2 was more associated to the ability to follow specific features of the pieces (i.e. rhythm).

Factor 1 revealed a significant interaction between Conductor and Piece using Wilks’ Lambda (F(4,6)=10.18; p=0.007) but no simple effects (Conductor: F(1,9)=0.14; p=0.72; Piece: F(4,6)=1.07; p=0.45). Similarly the Greenhouse-Geisser corrected RM ANOVA reported a significant interaction (F(4,36)=4.17; p=0.007; G-G: Epsilon=0.547; p_adj_=0.028) but no simple effects (Conductor: F(1,9)=0.14; p=0.71; G-G: Epsilon=1; p_adj_=0.71; Piece: F(4,36)=1.79; p=0.15; G-G: Epsilon=0.72; p_adj_=0.17). The interaction was further explored with follow-up tests, revealing a difference between conductors in pieces 3 (p=0.02) and 5 (p=0.009). Analyses on Factor 2 instead revealed no significant effects with both Wilks’ Lambda and the Greenhouse-Geisser corrected RM ANOVA.

**Caption**

Figure S1: Factor loadings for the subjective questionnaire items.

**References**

(30) Del Vecchio, D., Murray, R. M. & Perona, P. Decomposition of Human Motion into Dynamics Based Primitives with Application to Drawing Tasks. *Automatica* **39** (2003).

(31) Lu, C. & Ferrier, N. J. Repetitive motion analysis: segmentation and event classification. *IEEE Trans. Pattern Anal. Machine Intell*. **26**, 258-263 (2004).

(32) Bissacco, A. Modeling and learning contact dynamics in human motion. Proc. of the IEEE Computer Society Conference on Computer Vision and Pattern Recognition (CVPR), IEEE Computer Society, 2005.

(33) Bissacco, A. & Soatto, S. Classifying Gaits Without Contact Forces. Proc. of the IEEE Computer Society Conference on Computer Vision and Pattern Recognition (CVPR), IEEE Computer Society, 2006.

(34) Akaike, H. A new look at the statistical model identification. *IEEE Trans. Autom. Control* **19**, 716-723 (1974).

(35) Schwartz, G. Estimating the Dimension of a Model. *Ann. Stat.* **5**, 461-464 (1978).

(36) Ding, M., Chen, Y. & Bressler, S. L. in *Handbook of Time Series Analysis,* (Eds. Schelter, S., Winterhalder, N. & Timmer, J.) 437-460 (Wiley, Wienheim, 2006).

(37) Freiwald, W. A. *et al*., Testing non-linearity and directedness of interactions between neural groups in the macaque inferotemporal cortex *J. Neurosci. Meth*. **94**, 105-119 (1999).

(38) Chen, Y. H., Rangarajan, G., Feng, J. F. & Ding, M. Z. Analyzing multiple nonlinear time series with extended Granger causality *Phys. Lett. A* **324**, 26-35 (2004).

(39) Ancona, N., Marinazzo, D. & Stramaglia, E. Radial basis function approach to nonlinear Granger causality of time series. *Phys. Rev. E* **70**, 056221 (2004).
